# Potential Energy Landscape and Robustness of a Gene Regulatory Network: Toggle Switch

**DOI:** 10.1371/journal.pcbi.0030060

**Published:** 2007-03-30

**Authors:** Keun-Young Kim, Jin Wang

**Affiliations:** 1 Department of Physics and Astronomy, State University of New York Stony Brook, Stony Brook, New York, United States of America; 2 Department of Chemistry, State University of New York Stony Brook, Stony Brook, New York, United States of America; 3 State Key Laboratory of Electroanalytical Chemistry, Changchun Institute of Applied Chemistry, Chinese Academy of Sciences, Changchun, Jilin, People's Republic of China; University of North Carolina, United States of America

## Abstract

Finding a multidimensional potential landscape is the key for addressing important global issues, such as the robustness of cellular networks. We have uncovered the underlying potential energy landscape of a simple gene regulatory network: a toggle switch. This was realized by explicitly constructing the steady state probability of the gene switch in the protein concentration space in the presence of the intrinsic statistical fluctuations due to the small number of proteins in the cell. We explored the global phase space for the system. We found that the protein synthesis rate and the unbinding rate of proteins to the gene were small relative to the protein degradation rate; the gene switch is monostable with only one stable basin of attraction. When both the protein synthesis rate and the unbinding rate of proteins to the gene are large compared with the protein degradation rate, two global basins of attraction emerge for a toggle switch. These basins correspond to the biologically stable functional states. The potential energy barrier between the two basins determines the time scale of conversion from one to the other. We found as the protein synthesis rate and protein unbinding rate to the gene relative to the protein degradation rate became larger, the potential energy barrier became larger. This also corresponded to systems with less noise or the fluctuations on the protein numbers. It leads to the robustness of the biological basins of the gene switches. The technique used here is general and can be applied to explore the potential energy landscape of the gene networks.

## Author Summary

Cellular networks are at the heart of systems biology at present. To understand how cellular networks function in these highly fluctuating environments, a global approach is needed. Here we provide a global framework, in terms of potential landscapes, for studying the gene regulatory networks in the presence of the intrinsic statistical fluctuations. We uncovered the underlying landscape for the network. We identified the basins of attraction of the landscape as the biological functional states. The potential barrier between the two basins determines the time scale of conversion from one to the other. The robustness of the biological functional states of the network, the gene switches in this case, can be guaranteed if the conversions among the basins of attraction are not frequent, or, in other words, the barriers among the basins are relatively large. More detailed features of the network, such as the key genes or regulating links relevant to diseases (i.e., cancers), can be uncovered from the underlying landscape. Our technique is general and can be applied to explore the potential landscape of more realistic gene networks. Furthermore, our approach can also be helpful in guiding the network optimal design for synthetic biology.

## Introduction

In the post-genome era, with a wealth of data on genomic sequences, the crucial question becomes how to understand the organization of these sequences in nature and how genes function [1–4]. This is a challenging task. According to the central dogma, turning gene switches on and off controls certain types of protein synthesis and production. Furthermore, the on and off of gene switches determines the developmental plans of the cell. On the other hand, the protein products generated by the gene switches act back on the genes to control their expression patterns. The gene regulations thereby form a network with inherent many-body interactions and feedback loops. That is why the system often becomes quite complicated and hard to study.

The underlying nature of cellular networks has been explored by many experimental techniques [4]. It has often been found that cellular networks are in general quite robust and perform their biological functions in the midst of environmental perturbations. There have recently been an increasing number of studies on the global topological structures of cellular networks [5–8]. However, so far there are very few studies from the physical point of view of why the networks are so robust and why they perform their biological functions [9–20].

Theoretical models of cellular networks have often been formulated with a set of chemical reaction equations in bulk. These averaged dynamical descriptions are inherently local. To probe the global properties, one often has to explore different parameters. Since the parameter space is huge, the issue of global robustness is hard to address directly from these approaches.

Here we will explore the nature of the network from another angle: we formulate the problem in terms of the potential energy function or potential energy landscape. If the potential landscape of the cellular network is known, the global properties can be explored [21,22]. This is analogous to the fact that the global thermodynamic properties can be explored when knowing the inherent interaction potentials in a system.

There is another intriguing factor controlling the gene expression patterns. In the cell, there are a finite number of molecules (typically on the order of several hundreds or thousands). The intrinsic statistical fluctuations, usually not encountered in bulk due to the large-number averaging, can be significant and play an important role in the dynamics of gene expression. This gives the source of intrinsic statistical fluctuations or noise. On the other hand, the fluctuations from highly dynamical and nonhomogeneous environments of the interior of the cell give the source of the external noise for the networks [23–30]. It is important to investigate the roles of the statistical fluctuations or noises on the robustness and stability of the network.

**Figure 1:**
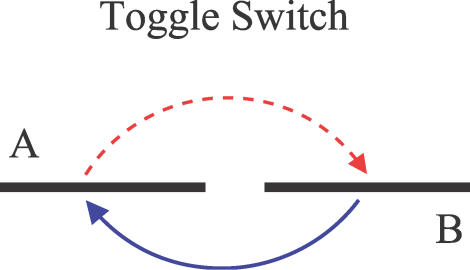
Toggle Switch: Protein A Represses Gene B (Dotted Line), and Protein B Represses Gene A (Solid Line)

In general, instead of studying the averaged chemical reaction network equations in bulk, we should use statistical descriptions to model the cellular process. This can be realized by constructing a master equation for the evolution of probability instead of average concentration for the corresponding chemical reaction network equations [26,31–35]. One can also study the steady state properties of these probabilistic chemical reaction network equations. The generalized potential energy for the steady state of the network can be shown to be closely associated with the steady state probability of the network in general [10–12,15–20,31,32]. Once the network problem has been formulated in terms of the generalized potential function or potential landscape, the issue of the global stability or robustness is much easier to address. In fact, some explicit illustrations of the potential energy landscape and robustness for the MAP Kinase signal transduction network and cell cycle have been given recently [19,20]. It is the purpose of this paper to study the global robustness problem directly from the properties of the potential landscape for a simple yet important gene regulatory network: a toggle switch. Figure 1 shows a toggle switch. Gene networks often involve many degrees of freedom. To resolve the issue of multidimensionality, instead of using the direct Monte Carlo simulation [33] for solving the master equations, a Hartree mean field approximation can be applied to reduce the dimensionality and address the global issues [11,12,36–38].

There are three aims of this paper. Our first aim is to develop a time-dependent Hartree approximation scheme [36] to solve the associated master equations to follow the evolution of multidimensional probability of the network. Our second aim is to construct the underlying potential energy landscape for a toggle switch [39] and explore both the steady state and time evolution of the landscape. Our third aim is to study the phase diagram of the system and the kinetic time scale from one stable basin of attraction to another in different conditions. We will address the global robustness condition for a toggle switch.

## Methods and Materials

As our goal is to uncover the potential energy landscape, we first studied the chemical reaction network involved in gene regulations. In particular we need to take into account the intrinsic statistical fluctuations due to the finite number of molecules in the cells. The statistical nature of the chemical reactions can be captured by the corresponding master equations. We established master equations for the gene regulations that describe the evolution of the networks probabilistically. The master equation is almost impossible to solve due to its inherent huge dimensions. We therefore used the Hartree approximation to reduce the dimensionality [11,12,36]. In this way, we could follow the time evolution and steady state probability of the protein concentrations. The steady state probability is closely associated with the underlying potential energy landscape, which is our ultimate target.

### Assumptions

Gene expression is regulated in various and complex ways, and can be represented by many coupled biochemical reactions. In this report, our goal was not just to explain some specific gene network system as accurately as possible, but to illustrate mathematical tools for exploring the general mechanisms of transcriptional regulatory gene networks. We therefore took abstractions of some essential biochemical reactions from complicated reactions of diverse systems.

Let us start with the explanation of some terminologies used in this manuscript: “activator” is a regulatory protein that increases the level of transcription, “repressor” is a regulatory protein that decreases the level of transcription. By “operator” we mean the DNA site or the gene where regulatory proteins (either an activator or a repressor) bind. First we are interested in the effect of “operator fluctuation” by which we mean the biochemical reactions that change the state of the operator. The operator is said to be in an occupied state if a regulatory protein is bound to it, and in an unoccupied state if the protein is not bound to it. For the repressor we include the following reaction.

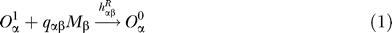


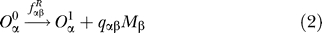
where 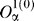 stands for the active (inactive) operator state of gene *α, M_β_* represents the regulatory protein synthesized or produced by gene *β,* and *q_αβ_* is for the multimer-type of proteins. For example, if *q_AB_* = 2(3), dimer (tetramer) proteins produced from gene B repress the expression of gene A, 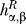 and 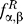 are reaction probabilities per unit time. In a similar way, we may also consider the activator:

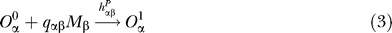


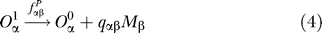



Notice that the superscript 1(0) in 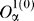 indicates the activity state of the operator and does not represent the bound state of regulatory protein. We will say the gene is on (off) when the operator of the gene is active (inactive). The gene will be on when it is occupied by activators or when repressors are unbound from it.

Next we include the transcription and translation steps. Here we ignore mRNA and consider only one step combining transcription and translation:

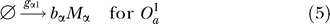


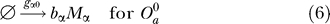


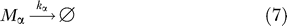
where ∅︀ denotes a protein sink or source, *b_α_* stands for the burst size of produced proteins (*M_α_*), *g*_1(0)_ is a protein synthesis probability per unit time, and *k_α_* is the degradation probability per unit time.

We can say that Equations 1–7 are “effective reactions” of the transcriptional regulatory gene network system. Roughly speaking, we can say the other biochemical reactions could be taken into account by adjusting the parameters of the reaction probabilities per unit time. In this sense, the reaction parameters are not really constants but functions of time. Furthermore, the proteins may not be well-mixed in the cell, and the number of proteins could be a function of position. So we can generalize this formalism in a space-dependent manner. We also can add more species and reactions to the master equations. In this report we will assume homogeneity of the number of proteins and ignore the time delay (for example, due to the translation process) so that all the parameters are constants. Now we can construct the master equation based on the above assumptions and chosen effective reactions.

### Hartree Approximation of the Master Equation

The master equation is the equation for the time evolution of the probability of some specific state *P*:

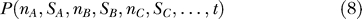
where A,B,C, … is the label of each gene; *n_A_*, *n_B_*, *n_C_*, … is the number of proteins expressed by gene A,B,C, … , respectively. *S_A_*, *S_B_*, *S_C_*, … is 1 or 0, and represents the activity state of the operator. The number of states, *N*, is *n_A_* × 2 × *n_B_* × 2 × *n_C_* × 2 × … . We expected to have *N*-coupled differential equations, which are not feasible to solve. Following a mean field approach [11], we used the Hartree approximation to split the probability into the products of individual ones: First, let us assume

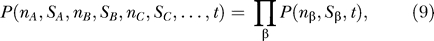
and sum over all indexes except one specific index that we are interested in, say α. This effectively reduces the dimensionality from exponential *n_A_* × *n_B_* × ··· *n_N_* × 2*^N^* to multiples (*n_A_* + *n_B_* + …) × 2 × *N,* and therefore the problem is computationally tractable. Finally we are left with two equations, one for *P*(*n*_α_, 1) and one for *P*(*n*_α_, 0). (In fact, these are not just two equations because *n*_α_ varies from 0 to hundreds.) With the two component vector notation,

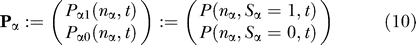
we have the compact form for the network:

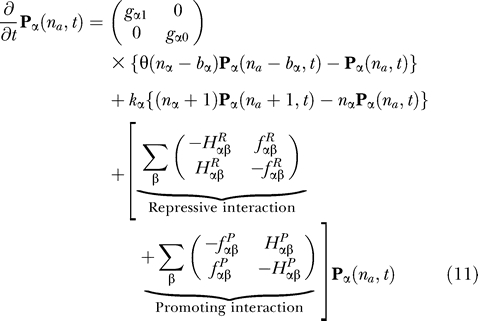
where

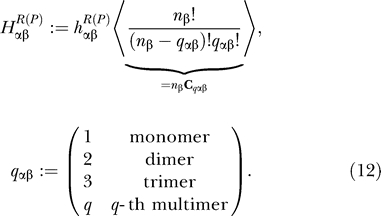



Notice that Equation 11 is simply a “birth–death” process without the last term. We will call the first two terms in Equation 11 the birth–death part or “drift and diffusion” part from the viewpoint of the diffusional Fokker–Plank equation [10,35]. Furthermore, we will call the last term the operator fluctuation part. In Equation 11, all other indices except *α* appear only in *H_αβ_* in the ensemble-averaged form (*f_αβ_* is just some number). If we deal with the one gene case, there is no ensemble average in Equation 12. The first effect of the operator fluctuation is the sum over *n_β_* and *S_β_*. The second effect is to cancel out many of the birth–death terms of other genes. Since α = *A*, *B*, *C*, … , we have the vector equation set of the same numbers as those of the genes. They are coupled to each other through the term *H*_αβ_. All network interactions can be determined by assigning every *h_αβ_*. *b_α_* is the number of proteins produced in bursts from gene *α,* and *θ* is a step function. In Equation 12, we take into account several kinds of binding proteins, and use proper combinatorics and ensemble average.

### Quantum Field Theoretic Description

The techniques of quantum field theory can be used to solve the master equation [11,37]. The first step is to construct a many-body quantum state. Notice that the probabilities defined by Equation 10 are imbedded in the quantum state as coefficients (Equation 14)

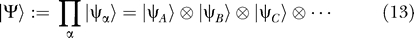
where

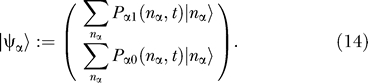
In Equation 13 we make an ansatz of Hartree-type product for the many-body state. Then non-Hermitian “Hamiltonian” of only repressive proteins, Ω, yields:

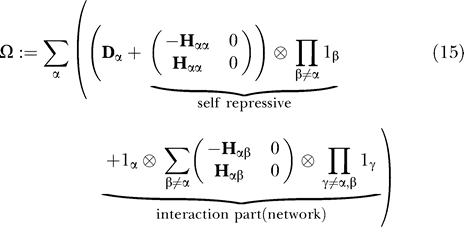
where

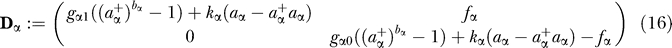


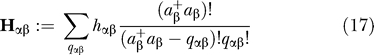



For each protein concentration, a creation and an annihilation operator are introduced, such that *a* ^+^|n〉 = |n + 1〉 and *a*|n〉 = n|n − 1〉. These operators satisfy [*a*, *a* ^+^] = 1. The generalization to include activating proteins is straightforward. While the state vector is a simple product of individual genes, the operator product form of Ω is chosen deliberately to reproduce the original master Equation 11. The Ω of a many-gene system seems to be Ω = Σ Ω*_i_* [11], but it would not be the simple sum of individual operators because of the interaction terms. Like the master equation, **D**_α_ is the birth–death part and plays a role in the diffusion and drift terms in the context of Fokker–Plank equation. The second term and third term in Equation 15 are repressor-related terms, and **H** *_αβ_* is the counterpart of *M_αβ_* in Equation 12. Finally, we have the following quantum system:

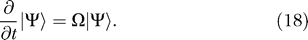



To complete the mean-field approximation, we need to average all interaction effects by doing an inner product with some reference state, which is a two-component generalization of the Glauber state [37]. If we are interested in an α-gene (operator) state and the associated protein, we may define the reference state:

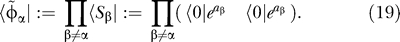
Then,

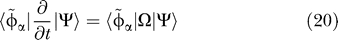
is equivalent to the master Equation 11.

### Solutions

#### Ritz's variational method with coherent state ansatz.

We will use the Rayleigh–Ritz variational method to obtain an approximate solution of a non-Hermitian Hamiltonian system (nonequilibrium system) like Equation 18 [11,38]. The master equation is equivalent to the functional variation δΓ/δΦ = 0 of an effective action Γ = ∫dt 〈Φ|(∂*_t_* − Ω)|Ψ〉. The method is analogous to the traditional procedure in quantum mechanics, except the modification due to non-Hermitian properties of the operator, of which left eigenvectors and right eigenvectors do not have to be the same. We will make a ket state ansatz, |Ψ〉, and a bra state ansatz, 〈Φ|, respectively [11]:

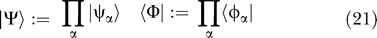


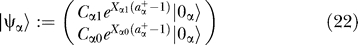


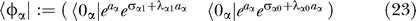
where *C_α_*_1_, *C_α_*_0_, *X_α_*_1_, *X_α_*_0_, α*_α_*_1_, α*_α_*_0_, λ*_α_*_1_, λ*_α_*_0_ are time-dependent parameters to be determined by the variational principle. The ket ansatz is chosen as coherent state, which corresponds to a Poisson distribution. *C_α_*_1_ and *C_α_*_0_ are the probabilities of the two DNA-binding states, *S* = 1 and *S* = 0, respectively. The coupled dynamics of the DNA-binding state and the protein distribution are described as the motion of wavepackets with amplitudes *C_α_*_1_ and *C_α_*_0_ as well as by means of the protein concentrations at *X_α_*_1_ and *X_α_*_0_ (from the Poisson distribution ansatz). With the following notation

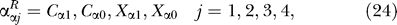
respectively α = *A, B, C,* …

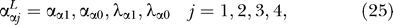
respectively α = *A, B, C,* … .

Here, 〈Φ|(α*^L^* = 0) is set to be consistent with the probabilistic interpretation 〈Φ(α*^L^* = 0)|Ψ(α*^R^*)〉 = 1. The condition for the extremum of the action with respect to 〈Φ| is:

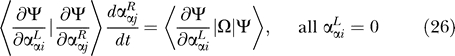
which is reduced to the coupled ordinary differential equations with parameters:

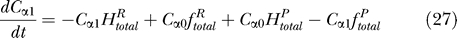


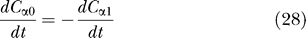


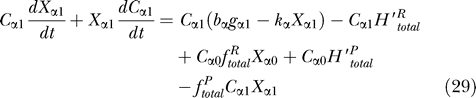


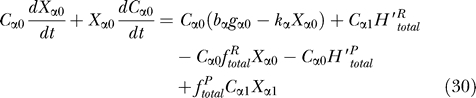


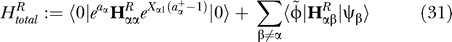


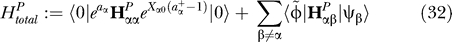


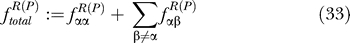


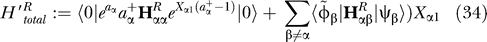


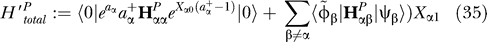
where 〈 ϕ~_β_| is defined by Equation 19. We can interpret Equation 27 as a master equation of probabilities of active operator states related to reactions Equation 1–4. However its change of the rate is based on the average number of proteins (Equations 31–32) unlike usual master equations which deal with the specific state. Equation 28 is just probability conservation. The first term of Equation 29 and Equation 30 is related to protein synthesis and degradation (Equations 5–7), and the rest are the effects of operator fluctuations.

Equations 27–30 are the general equations for parameters; we can use these equations for any number of gene systems and for any kind of regulatory patterns by assigning the binding and unbinding probability rate.

#### Moments equation.

The mean-field approximation approach should inherently provide information on moments (Equation 12), so it is natural to construct moment equations from Equation 11 [12]. The k-th moment of the protein number is obtained by the following equations. The 0-th moment of protein number is interpreted as the probability that gene is on or off. We also derived the moments equations from Equation 18

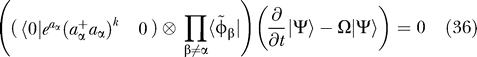


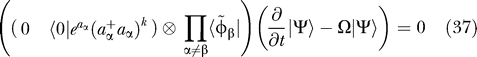
In principle, once we know all the moments, we can construct a probability distribution; but in many cases, we cannot get all the moments. However, Equations 36 and 37 have a good structure if there is no self-interaction, since all the moments can be computed by some lower moments recursively. If we deal with *q*-th multimer proteins, we can get all the moments higher than *q* from 0-th, . . . ,*q*-th moments. In the toggle switch and the dimer protein cases, for example, we have 12 independent equations (3 × 2 × 2: (*k* = 0, 1, 2), (α = A,B) for each Equation 36 and Equation 37) and 12 variables (*C*_α1_, *C*_α0_, 〈*n*_α1_〉, 〈*n*_α0_〉, 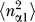 , 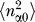 , α = *A*, *B*); we show these explicitly in Equations 45–50, where we used two constraints of probability conservation, so there are only ten equations. We can say these equations are closed. However, if we include self-interaction, the equations are not closed, so they cannot be solved exactly [12].

#### Relation between the ansatz equation and the moments equation.

Equations 27–30 can be derived from Moment Equations 36 and 37 without using the variational principle. We can start from moment equations and then assume a specific probability distribution based on a physical argument, which gives some specific relations between moments. For example, the Poisson distribution has only one parameter, so we may calculate all other moments from the first moment, the mean. Moment equations with a Poisson ansatz give us the same equations as the variational approach in Equations 27–30.

Moment equations are more exact than the variational approach, but the approach cannot be used to obtain exact solutions for the system having self-interaction, in which equations are not closed. Even in the closed system, an ansatz reduces the degrees of freedom significantly and makes the problem easier to handle. Mathematically, using an ansatz is equivalent to giving specific relations between moments. We may, therefore, not need to take care of higher moments if an infinite number of higher moments is automatically given by assuming a specific ansatz. In practice, ansatz might be useful. Then the issue would be how faithful the ansatz we choose is. In this paper we used both the moment equation and the Poisson ansatz. Notice that Equation 11 is merely a birth–death process without the last term. In the limit that the last term is small enough, the so-called adiabatic limit (faster protein number fluctuations compared with the DNA state alterations), we expect the solution will be close to the Poisson distribution.

### Interpretation of the Solutions

The final output we get from these equations is basically moments. From these moments we need to construct the total probability. There are several important features to be pointed out. We start with the single gene case.

First, notice that the total probability does not have the structure of *C*_1_ *P*_1_ + *C*_0_ *P*_0_. We started with a two-component column vector and to extract the physical observables we needed to do the inner product with a two-component row state vector. (We never added the spin up and down component directly in quantum mechanics.) The total probability should therefore not follow the steps of constructing *P*_1_ and *P*_0_ first and then weighing by *C*_1_ and *C*_0_. The correct procedure is the following. With the moments, the solutions of equations, we construct new moments:

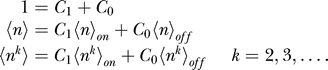



In principle, we can get arbitrary order of moments and construct the corresponding probability if the equations are closed. In practice, however, we may choose one of two probability distributions: Poisson or Gaussian distributions.

Second, the probability obtained above corresponds to one limit point or basin of attraction. One solution of the equations determines one of the limit points and also gives the variation around the basin of attraction, so it is intrinsic. If the system allows multistability, then there are several probability distributions localized at each basin of attraction, but with different variations. Thus, the total probability is the weighted sum of all these probability distributions. The weighting factors (*w^a^*, *w^b^*) are the size of the basin, which is nothing but the relative size of the set of initial values ending up with a specific basin of attraction.

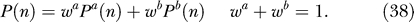



Notice that the steady state solution is not enough to describe the total probability. It does not say anything about the volume of the basin, it only tells us the limit point. So the effort to derive an effective potential energy from the steady state solution on general grounds needs to take into account the volume of the basin of attraction. One simple exception is the symmetric toggle switch, where the weighting factors are simply (0.5, 0.5) by symmetry.

Third, the total probability of many genes is simply the product of each gene based on our basic assumption, the mean field approximation. For example, the probability of a toggle switch can be written as

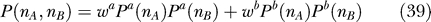
where *a* and *b* denote each limit point, and *w^a^* and *w^b^* are the weighting factors. Even though it is simply multiplication, the interactions between them are already taken into account from the coupled equations.

Finally, once we have the total probability, we can construct the potential energy (or potential energy landscape) by the relationship with the steady state probability:

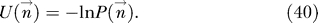



This is the reverse order of the usual statistical mechanics of first obtaining the potential energy function, exponentially Boltzman weighting it, and then studying the partition function or probability of the associated system. Here we look for the inherent potential energy function from the steady state probability. In the gene-network system, every chemical parameter, such as the protein production/decay rates and binding/unbinding rates, will contribute to the fluctuation of the system. All these effects are encoded in the total probability distribution, and, consequently, in the underlying potential energy landscape.

## Results

We looked at an important example of two genes interacting with each other. The interactions are through the proteins synthesized by the genes, which act back to regulate the gene switch. The bacterial lambda phage is a good biological example of a toggle switch. The two lysogenic and lysosic genes are both stable and robust. It has been a long-standing problem to explain why the lambda phage is so stable [11,12,16,34,40]. We addressed this issue in the presence of the intrinsic statistical fluctuations of the finite number of the proteins in the cell by exploring the underlying potential energy landscape. First we solved the master equation to obtain both the time-dependent and steady state probability distributions of the protein concentrations of the corresponding genes. Then we inferred the underlying potential energy landscape from the steady state probability distribution of the protein concentrations. We then considered the symmetric toggle switch.

All applications to specific network systems start with Equations 27–30. First, we chose the number of genes and designed the interaction type (network topology) and protein types (multimers). Second, we assigned the strength of the parameters. Then we solved this coupled ordinary differential equation system numerically with certain initial conditions. We considered a toggle switch [39] of two genes, as shown in Figure 1, which has wide application in molecular biology, such as the bacteriophage λ problem [34]. Let us start with the toggle switch case.

### Symmetric Toggle Switch

For the symmetric switch, we first solved the equations of motion determining the amplitude, the mean, and the higher order moments of the probability distribution of the protein concentrations of the corresponding genes. These are given below.

### Poisson and Moment Equation Solutions of Master Equations

We solved the master equation with two methods. One is the Poisson ansatz, mentioned above, by assuming the inherent Poisson distribution, and the other is the exact method, using the moment equation. For the inherent Poisson distribution, we can write down the equations of motion for the amplitude and mean.

#### Poisson ansatz.



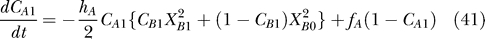


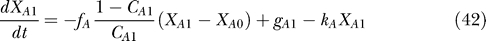


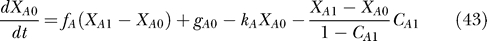


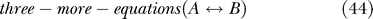
where we eliminated two variables by the probability conservation (*C*_α1_ + *C*_α0_ = 1), and recollected terms.

For the exact solution with moment equations, we also wrote down the equations of motion of the moment of protein concentration of the corresponding genes.

#### The corresponding moment equations.



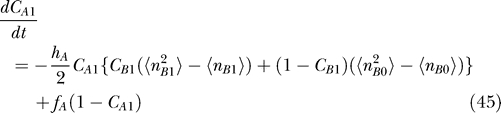


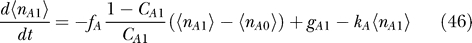


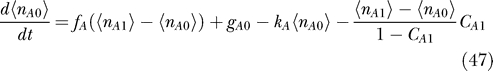


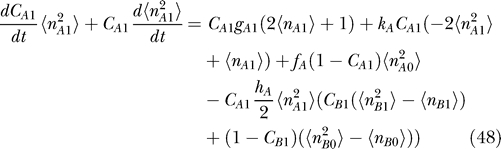


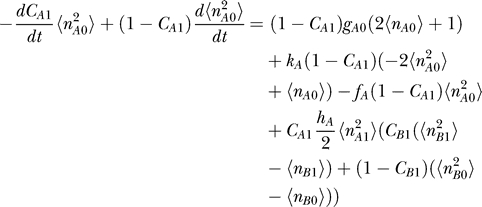


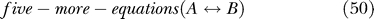



### Monostability versus Bistability for Symmetric Toggle Switch

**Figure 2:**
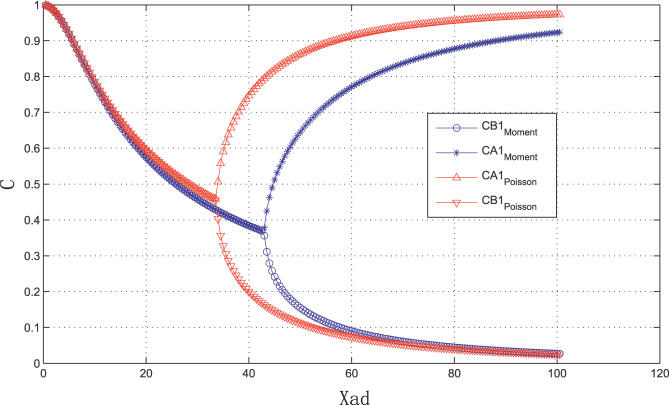
Probability C That Genes Are in the Active State as a Function of *X_ad_* = (*g*_1_ + *g*_0_)/2*k_A_* for a Symmetric Switch Showing the Bifurcation Exact moment equation solutions are compared with Poisson ansatz solutions 0 < *X_ad_*(= *g*_1_/2) < 100 , *k_A_* = *k_B_* = 1, *f_A_* = *f_B_* = 0.5, *h_A_* = *h_B_* = *f_A_*/500, and *g*_A0_ = *g*_B0_ = 0.

By giving some initial conditions, and taking the long time limit, we obtained the steady state solution. We fixed all parameters except the protein synthesis rate *g*_A1_(= *g*_B1_). We looked at the probability of genes that were in the active state versus the relative importance of synthesis rate versus degradation rate. By increasing the synthesis rate, *g*_A1_, we could observe the bifurcation from the monostable state to the bistable state after passing a certain critical point. Figure 2 shows the result of taking the long time limit of the equations of motion. The two curves (with subscript Moment) are from moment equations, and the others are from the Poisson ansatz. This is consistent with the results directly from time-independent equations (Figure 3A in [12]). We used the parameter *X_ad_* = *ḡ*/*k* as the horizontal axis variable. It is simply *g*_1_/2 in our choice.

**Figure 3:**
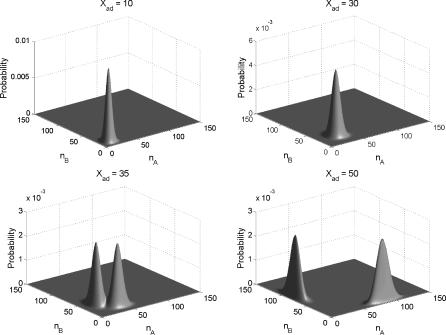
Steady State Probability of Symmetric Toggle Switch (Long Time Limit) as a Function of the Number of Protein A and the Number of Protein B for Different *X_ad_* = *g*_A1_/2*k_A_* The other parameters are the same as Figure 2.

In the parameter range in which the bistability occurs, we found two limit points (named *a* and *b*) in the numerical analysis. Now from the solution of the equations we constructed the probability of protein numbers or concentrations (all illustrations in this paper were based on the Poisson ansatz for simplicity, but it can be easily done with the moment equations and qualitative features will not be changed),

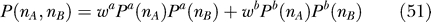



For the symmetric toggle switch case, the weight factor was simply (0.5, 0.5) due to symmetry. The change of the probability distribution shape in terms of the adiabatic parameter of the relative importance of the protein synthesis rate compared with the degradation rate is shown in Figure 3. These figures show the monostability to bistability of the symmetric toggle switch. For a large enough protein synthesis rate relative to degradation rate, bistability emerges.

### Potential Energy Landscape: Monostability to Bistability

**Figure 4:**
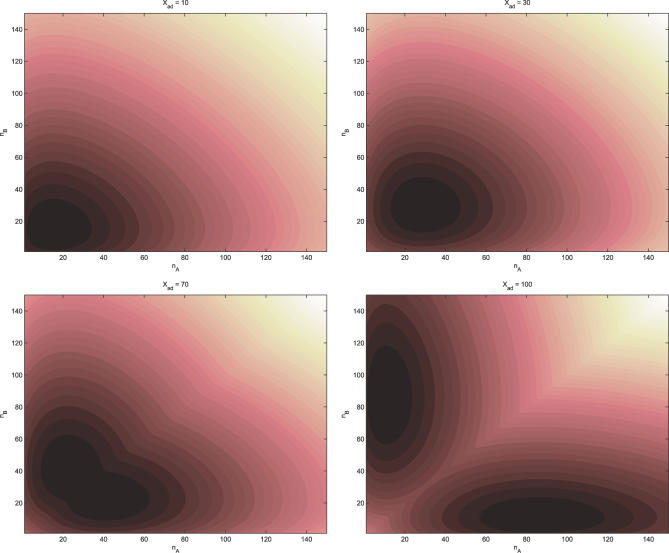
Potential Energy of Symmetric Toggle Switch as a Function of the Number of Protein A and the Number of Protein B for Different *X_ad_* = *g*_A1_/2*k_A_* The other parameters are the same as Figure 2.

As we discussed, the steady-state distribution function *P*↛ for the state variable *↛* can be expressed to be exponential in a function *U↛*:

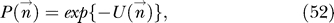
where *P↛* is already normalized. From the steady state distribution function, we can therefore identify *U* as the generalized potential energy function of the network system. In this way, we map out the potential energy landscape. Figure 4 shows the potential energy landscape corresponding to Figure 3.

**Figure 5:**
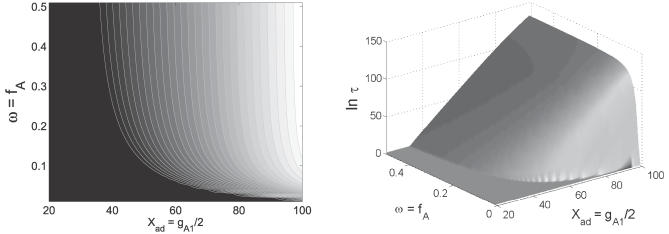
The Time Scale of the Transition between the Two Stable Minimum Basins of Attractions as a Function of ω = *f_A_*/*k_A_* and *X_ad_* = *g*_A1_/2*k_A_* The other parameters are the same as Figure 2.

We can see that when the protein synthesis rate is small relative to degradation rate, only a single basin of attraction exists for the underlying potential energy landscape. For large enough protein synthesis rate relative to degradation rate, two basins of attraction emerge. Once we have the potential energy landscape, we can discuss the global stability of the gene regulatory networks. The time scale of the transition between the two stable minimum basins of attraction can be estimated by τ ∼ τ_0_exp[*U* ^≠^ − *U_min_*] [41]. Here, τ_0_ is the pre-factor and τ is the time scale of transition from one basin of attraction to the other. *U* ^≠^ is the potential energy at the saddle point between the two stable basins of attraction. *U_min_* is the potential energy at one of the basins of attraction. Thus *U* ^≠^ − *U_min_* represents the potential energy barrier height between two stable basins of attraction. In Figure 5 we can see that as the synthesis rate and unbinding rate of protein to DNA increase relative to the degradation rate, the potential barrier height between the two basins of attraction increases. The time scale of the transition from one basin of attraction to the other exponentially increases with the barrier height.

Figure 5 shows the phase diagram of the parameter ranges for the monostable basin and two bistable basins of attraction. We can see that when the synthesis rate and unbinding rate of protein to DNA are low relative to the degradation rate, the potential energy landscape prefers one stable basin of attraction. As the synthesis rate and unbinding rate of protein to DNA increases relative to the degradation rate, the potential energy landscape gradually develops the two stable basins of attraction from the monostable one. There is a transition from monostable to bistable basins of attraction of the underlying potential energy landscape at certain parameters.

This illustrates how biological robustness is realized for the toggle switch. As the protein synthesis rate and unbinding rate of protein to DNA increase relative to the degradation rate, more proteins are synthesized. These proteins are strong repressors. This leads to smaller fluctuations. Furthermore, the associated barrier height between the two basins of attraction becomes large, and the two basins of attraction become more stable since it is harder to go from one well to another. So, small fluctuations and large barrier heights both serve as the source for the robustness and stability of the gene toggle switch. In other words, it is more unlikely for the system to change from one basin of attraction to the other. Therefore, the system becomes robust. The robustness issue is not yet well-understood for cellular networks in general. Here we explored the robustness of the switches against the intrinsic statistical fluctuations coming from the finite number of protein and DNA molecules. This is clearly very important and has potential applications to the robustness problem of lambda phage in bacteria.

### Time Evolution of the Underlying Potential Energy Landscape

**Figure 6:**
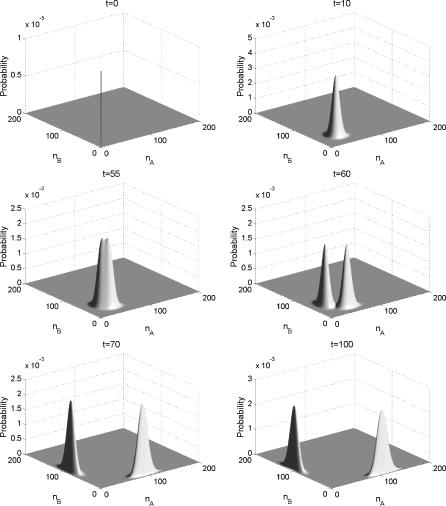
The Time Evolution of the Probability of Symmetric Toggle Switch as a Function of the Numbers of Protein A and the Numbers of Protein B *X_ad_* = 60, and the other parameters are the same as Figure 2. *t* = 0, 10, 55, 60, 70, 100.

**Figure 7:**
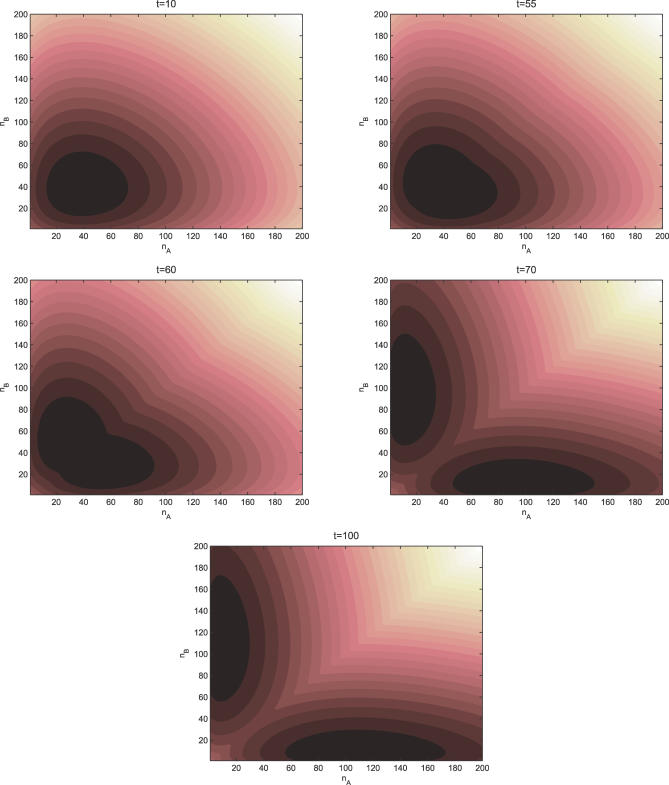
The Time Evolution of the Potential of Symmetric Toggle Switch as a Function of the Numbers of Protein A and the Numbers of Protein B *X_ad_* = 60, and the other parameters are the same as Figure 2. *t* = 10, 55, 60, 70, 100.

We also studied the time evolution of the probability and the potential energy landscape with dynamic equations. We chose the specific parameters and initial conditions to illustrate the idea. The results are shown in Figures 6 and 7.

In Figures 6 and 7, we see the evolution in time of the probability and the underlying potential energy landscape from the flat land at the beginning to the full development of two basins of attraction at the steady state. This is the first illustration of the dynamical evolution or formation of development of the potential energy landscape of a toggle switch.

## Discussion

Finding the multidimensional potential energy landscape is the key to addressing important global issues such as the robustness of cellular networks. We have uncovered the underlying potential energy landscape of a simple gene network: toggle switch. We found that as the protein synthesis rate and the unbinding of protein to DNA rate relative to degradation change from small to large, the underlying potential energy landscape changes from having monostable to bistable basins of attraction. These basins correspond to stable, biologically functional states. The potential barrier between the two basins determines the time scale of conversion from one to the other. We found that as the protein synthesis rate and unbinding of protein with DNA rate relative to degradation became greater, the potential energy barrier became greater and the statistical fluctuations were effectively more severely suppressed. This leads to the robustness of the biological basins of the gene switches.

In principle, our approach can be generalized to more realistic networks involving multiple genes as well as additional levels of regulations. This could be realized by averaging the interactions among genes in the corresponding master equations. It effectively reduces the dimensionality of the problem from exponential to polynomial number of degrees of freedom. It is worthwhile to note the limitation of this approach. When the interactions among genes are very strong, our approach is less effective.

Recently, synthetic biology became an important part of systems biology [42–45]. There has been significant progress in this field. However, there still seems to be a lack of general principles and algorithms guiding the design and construction of synthetic gene networks. The robustness condition (see Figure 5) found in this study would help us to identify the parameter and connectivity region to reach global robustness and function of the network. The optimal network design will be based on that. Furthermore, we can vary the parameters and connections to design different distinct features while maintaining the stability of the network.

The adaptive landscape idea was first introduced into biology by S. Wright in the 1930s [46–49]. Landscape construction for one dimension is rather straightforward. However, even the two-dimensional case becomes nontrivial. The recent efforts to understand global systems biology need the concept of landscape. Progress was made towards this from the dynamic system point of view, where the nontrivial nature of low-dimensional systems was illustrated [20,36,50]. There are still conceptual and methodological issues remaining for high dimensional systems. The stochastic method introduced here may pave the road towards solving this problem.

This model can be modified to include more biochemical reactions. To investigate the role of mRNA, we can consider the transcription and translation process separately. To focus on the statistical fluctuations of genes turning on and off, it is possible to generalize the formalism to compute the statistical fluctuations quantitatively. We also can take into account the spatial variation of the state variables, such as the number of proteins.
